# Work Values of University Students in Chinese Mainland, Taiwan, and Hong Kong

**DOI:** 10.1007/s10447-012-9155-7

**Published:** 2012-06-19

**Authors:** Shui Wai Wong, Mantak Yuen

**Affiliations:** 1 Department of Applied Social Sciences, Hong Kong Polytechnic University, Hung Hom, Hong Kong, China; 2 Center for Advancement in Inclusive and Special Education, Faculty of Education, University of Hong Kong, Pokfulam Road, Hong Kong, China

**Keywords:** Work values, University students, Chinese, Hong Kong, Taiwan, Mainland China

## Abstract

Leuty and Hansen (Journal of Vocational Behavior 79:379–390, 2011) identified six domains of work values in undergraduate students in the West. The review undertaken in this paper suggests that the factor structure of work values of university students in Mainland China, Taiwan and Hong Kong essentially matches these six domains, except for the omission of ‘Family Maintenance’ and Wang’s (Indigenous Psychological Research in Chinese Societies 2:206–250, 1993) ‘Instrumental Values.’ This suggests some commonality in the work values construct between the East and West, but there are a few subtle differences. It is argued that such differences heighten the need for measurement scales with context-specific and society-specific items when examining work values in different settings.

## Introduction

In all parts of the world today, ‘work’ represents an indispensable part of life for most people. To be ‘in work’ (employed) means much more than just earning money. Work fulfills a wide range of personal and family needs (economic, social, psychological) and provides people with a purpose in life (Nord, Brief, Atieh and Doherty 1990). Individuals, therefore, attach particular values to most aspects of work, and these values tend to influence how they choose specific career paths and how they feel about the work they engage in every day.

Work behaviors and related outcomes, such as attitudes, decision-making, commitment, satisfaction, performance, productivity and achievement, have increasingly attracted the attention of researchers, managers and career counselors. Studies have shown that ‘work values’ are potential predictors for such work behaviours and outcomes, as well as serving as an indirect index of work motivation (O’Brien 1992). Due to the importance of work values to individuals and the community, research on this topic is ongoing. Investigators have noted, however, that there are problems in researching this construct―particularly the fact that many facets are unobservable. There are also difficulties in measurement, in that subjectivity can affect observers, and work values are often confused with other constructs or phenomena (Hitlin and Piliavin 2004).

## Problems Encountered in Work Values Research

Basically, there is a lack of a commonly agreed definition of ‘work values’ (Dose 1997) and no consensus on their precise nature (Meglino and Ravlin 1998). Also, the relationship between ‘general values’ (or ‘life values’) and ‘work values’ is not free from controversy. Some researchers, including Zytowski (1994), consider work values simply as a sub-system of general values, but Lyons, Duxbury and Higgins (2006) argue that general values and work values are separate but related constructs. Attempting to integrate diversified theoretical conceptions, Roe and Ester (1999) suggested three conclusions in regard to the relationship between general values and work values; namely that both share a similar cognitive structure, that general values project into the work domain and produce the latter, and that the latter generalize into wider social life. Obviously, further research is necessary to establish the exact relationship between these two constructs.

In the same way that general values are often confused with other concepts, so too work values are easily confused with concepts such as beliefs, needs, goals, attitudes, interests, motivations, and personality type (Meglino and Ravlin 1998; Rottinghaus and Zytowski 2006). Pryor (1982) has even proposed that ‘work values’ is a poorly formulated and confused concept, and that the term should be replaced by ‘work aspects preferences.’ Overall, there is a lack of consensus on the structure of work values, and there are still difficulties in accurately conceptualizing the construct. It deserves future investigation.

With differing concepts of work values it is not surprising that different instruments have been developed to measure the construct, including the *Work Values Inventory* (WVI) (Super 1970), the *Minnesota Importance Questionnaire* (MIQ) (Lofquist and Dawis 1971), the *Work Aspect Preference Scale* (WAPS) (Pryor 1981), the *Personal Values Questionnaire* (England 1967), and the *Survey of Work Values* (Wollock, Goodale, Wijting, and Smith 1971). While the content of these instruments overlap to a certain extent, they are certainly not identical in scope, purpose or underlying constructs.

## Work Values of University Students in the Greater China Region

University students are considered to be in a developmental stage of ‘emerging adulthood’― a stage between adolescence and adult life. This is the period for formation of personal identity, and also of career decision-making (Hunter, Dik, and Banning 2010). Due to the close relationship between work values and career choices, the study of work values in university students becomes particularly relevant and important. Also, as university students will often eventually fill positions in companies and organizations, research on work values of university students has major implications for human resources personnel (Ma 2005). In the West, research on college students’ work values is abundant and readily available (cf., Duffy 2010; Duffy and Sedlacek 2007; Ng and Sears 2010; Pascual 2009; Ryckman and Houston 2003; Shaw and Duys 2005). In the East there has been rather less research, and at this stage there is a need to synthesize extant studies of work values, particularly in Chinese communities.

Mainland China, Taiwan and Hong Kong have been referred to as “Two Shores, Three Places” (兩岸三地). In spite of sharing the same cultural heritage, the three places have been ‘semi isolated’ for some time, and have developed their own peculiar social and political arenas and agendas. Similarities and differences can be identified across the three places in terms of social, economical and political aspects. Mainland China has been ruled by the Communist Party since 1949, whereas Taiwan has developed a democratic political system with two dominant political parties―the Chinese Nationalist Party and the Democratic Progressive Party. In contrast, Hong Kong was a British colony from 1842 until China resumed sovereignty in 1997. With this historical background, Hong Kong has developed a unique value system that combines both Chinese and Western cultures and attitudes, and provides a typical example of ‘crossvergence’ (Priem, Love and Shaffer 2000).

There are obvious differences between Mainland China, Taiwan, and Hong Kong. Confucianism, for instance, had been seriously attacked in Mainland China during the Cultural Revolution that occurred between 1966 and 1976, with this having an effect upon all aspects of life there, including how people regarded work and its purposes. In the separate case of Hong Kong, it has been deeply affected by Western culture and Western attitudes to business and work during its many years as a British Colony. As for Taiwan, Confucianism has been playing an important role in shaping people’s values and work behaviours (Chu 2008).

## Assessing Work Values

Leuty and Hansen (2011) identified six key domains of work values in undergraduate students at a university in the United States, based on a principal components analysis of the *Minnesota Importance Questionnaire* (MIQ), *Manhardt’s Work Values Inventory* (MWVI) and *Super’s Work Values Inventory-Revised* (SWVI-R). The six aspects comprise: (1) ‘Working Environment’ (e.g., physical conditions of the workplace); (2) ‘Competence’ (e.g., ability to rise to challenging work); (3) ‘Status’ (e.g., prestige associated with the work); (4) ‘Autonomy’ (e.g., independence in work situation); (5) ‘Organizational Culture’ (e.g., fair company policies); and (6) ‘Relationships’ (e.g., relationships with co-workers).

The purpose of this paper is to examine studies of university students’ work values in Mainland China, Taiwan and Hong Kong, and to compare the findings across these cultures, as well as with findings in the West.

## Work Values of University Students in Mainland China

The *PsycINFO database* yielded 23 results when searched in September 2011 using the keywords ‘work values’, ‘university students’ and ‘China.’ Further examination revealed, however, that only one article among these results was actually addressing specifically the topic of university students’ work values. Via the *China Journals Full-text Database* and wider searching, though, it was possible to locate some relevant additional empirical studies conducted in Mainland China. Nearly all the studies under review were of a quantitative or cross-sectional nature, with longitudinal and qualitative studies being rare.

Regarding the measurement of work values in these studies, some researchers simply adopted translated versions of the well-established scales developed in the West, including Super’s *Work Values Inventory* (1970). Some used localized scales developed in Taiwan, such as the *Work Values Scale* (Wu, Lee, Liu & O, 1996), while others developed their own instruments to fit local research subjects and context (e.g., Gao 2009; Hong and Wu 2007; Lu and Liao 2008; Zhang 2005).

For this review, only empirical studies using purpose-constructed scales were selected for investigation. These scales had usually been analyzed by exploratory factor analysis (EFA), and different factor models resulted ranging from 3 to more than 8 factors (refer to Table 1). A few studies adopted confirmatory factor analysis (CFA) to verify proposed work values structures or models (cf., Huang 2009; Jin and Li 2005; Tian, Xu, and Ruan 2004; Zhang, Li, and Wang 2011).

**Table 1:** Factor-models derived from empirical studies of work values of university students in Mainland China

Study & its nature	Research subjects/Respondents	Measures & statistical analysis	Work values domains of university students in PRC*
Zhang, Li, and Wang (2011)	N = 478 undergraduate students from 7 universities in Beijing	81-item self-constructed scale; EFA & CFA	1. Welfare
			2. Purpose (status, personal development, family maintenance, societal advancement)
			3. Criteria (right, societal, personal)
CS/Q			4. Software Resources
Gao (2009)	N = 377 undergraduate students from 8 universities in PRC	33-item self-constructed scale; EFA	1. Security for Material Rewards & Advancement
			2. Social Status & Reputation
CS/Q			3. Ability Utilization & Personal Development
			4. “Soft” Work Environment
			5. Autonomy & Flexibility
			6. Professions & Family
			7. “Hard” Work Environment
Huang (2009)	N = 712 undergraduate students from 10 universities in Beijing	50-item self-constructed scale; EFA & CFA	1. Self Development
			2. Security
CS/Q			3. Interpersonal Relationships
			4. Organizational Culture & Management
			5. Status & Reputation
			6. Altruism
Lu and Liao (2008)	227 undergraduate students from 8 universities in China	29-item self-constructed scale; EFA	1. Development
			2. Family
CS/Q			3. Materials Reward & Security
			4. Organizational Reputation & Advancement
			5. Interpersonal Relationship
			6. Status
			7. Organizational Reputation
			8. Comfortable Life
Meng (2007)	425 undergraduate students from 10 universities in Beijing	43-item self-constructed scale; EFA	1. “Soft” Work Environment & Personal Development
			2. Social Status & Reputation
CS/Q			3. Work Environment (Hardware) & Security
			4. Intrinsic Values
			5. Opportunity for Growth and Contribution
			6. Authority
			7. Welfare & Security
			8. Freedom & Economic rewards
Zhao and Zhang (2005)	280 student teachers from a university in Liuling	18-item self-constructed scale; EFA	1. Security & Contribution
			2. Status & Reputation
CS/Q			3. Autonomy& Development
			4. Economic Rewards
			5. Sense of Achievement
Wang, Ma, and Yao (2003)	undergraduate students from 4 universities in Beijing	70-item self-constructed scale; EFA	1. Materials Rewards & Environment
			2. Personal Growth & Development
CS/Q			3. Organizational Culture & Management
			4. Social Status & Organizational Development
Hong and Wu (2007)	1408 undergraduate students in Zhejiang	36-item self-constructed scale; EFA	1. Material Rewards
			2. Security
CS/Q			3. Organizational Culture
			4. Relationship
			5. Development
			6. Prestige
Yan and Gu (2007)	178 undergraduate students in Henan and Nanjing	20-item self-constructed scale; EFA	1. Development
			2. Hygiene Factor
CS/Q			3. Reputation of Work Unit/Company
			4. Prestige
Zhang, Wang, Yang, and Teng (2007)	661 Chinese college students from ten universities in China	Chinese college students' Work Values Questionnaire (CWVQ) (32 items): EFA	1. Challenge
			2. Personal Worth
CS/Q			3. Equitable Opportunity
			4. Social Status
			5. Personal Development
Wang and Kang (2005)	150 undergraduate students in Lanzhou	48-item self-constructed scale; EFA	1. Salary & Benefits
CS/Q			2. Self-Actualization
			3. Personal Interests
			4. Work Environment
			5. Background Variables
			6. Societal Need
			7. Job Stability
			8. Social Status
Jin and Li (2005)	813 undergraduate students in Beijing	34-item self-constructed scale; EFA & CFA	*Terminal Values*
			1. Family Maintenance
CS/Q			2. Status
			3. Achievement
			4. Societal Progress
			*Instrumental values*
			1. Stability
			2. Interests
			3. Norms & Morality
			4. Pay & Reputation
			5. Career Development
			6. Welfare & Benefits
Zhang (2005)	791 student teachers in Inner Mongolia	26-item self-constructed scale; EFA	1. Development
			2. Status
CS/Q			3. Prestige
			4. Welfare
			5. Work Environment
			6. Job Stability
			7. Family
Tian, Xu, and Ruan (2004)	804 undergraduate students in Lanzhou	12-item self-constructed scale; EFA & CFA	1. Prestige
			2. Opportunity & Stage for Performance
			3. Security
CS/Q			
Ling, Fang, and Bai (1999)	408 undergraduate students from 6 universities in China	22-item self-constructed scale EFA	1. Prestige
			2. Hygiene Factor
			3. Development
CS/Q			

*People’s Republic of China

*CS/Q* quantitative and cross-sectional study; *EFA* exploratory factor analysis; and *CFA* confirmatory factor analysis

The difference in number of factors identified across studies may be due to variations among the participating subjects, the method used to measure work values, sampling methods, and other research procedures (Zhao and Zhang 2005). The difference may also be because some factors embrace broad categories that seem to include diverse items. In addition, some factors, in spite of being given different names or labels, actually include similar items from the measurement scale. For example, Gao’s (2009) factor termed ‘Security for Material Rewards and Advancement’ and Zhang, Wang, Yang and Teng’s (2007) factor ‘Equitable Opportunity’, though labelled differently, both include items such as ‘fair competition’ and ‘equitable opportunity.’ To add to the potential confusion, some factors with the same name in different studies were derived from totally different items. For example, Huang’s (2009) factor ‘Interpersonal Relationship’ comprises items such as ‘social status’, ‘opportunity for decision making’ and ‘personal influence’, whereas Hong and Wu’s (2007) factor ‘Relationship’ comprises items such as ‘simple interpersonal relationships’, ‘collaboration with co-workers’, ‘development prospects’, ‘norms and culture of the organization’, and so on. It can also be noted that ‘Equitable Opportunity’ in Zhang *et al*.’s (2007) study is presented as a distinct factor in its own right; however, closer examination shows that ‘equal opportunity for fair competition’ appears merely as one item among many within the work values scale in various studies (cf., Gao 2009; Huang 2009; Ling, Fang, and Bai 1999; Lu, and Liao 2008; Meng 2007; Tian, Xu, and Ruan 2004; Wang and Kang 2005).

With locally designed or adapted scales, some of the items are context-specific. For example, issues that university students in Mainland China are most concerned with include: residence permits (*“hukou”*), couples working in the same city, insurance, and housing benefits. Items covering these issues appear in purposefully constructed work values scales in this location but are rarely found in other instruments.

Careful examination revealed that several factors or key domains consistently appeared in the factor structures of work values of university students in Mainland China. These key domains included: (1) Welfare and Security for Material Rewards/Comfortable Life (pay, fringe benefits, housing, insurance, job security, residence permits); (2) Status and Reputation (social status, reputation of the organization/company, opportunity for traveling abroad); (3) Ability Utilization and Personal Development (competency, challenging work, creativity); (4) Interpersonal Relationships (relationships with co-workers and supervisors); (5) Family Maintenance (taking care of parents, working with spouse in the same city, work-family balance, family expectations and influences); (6) Organizational Culture and Management (work culture, leadership, fair competition, equal opportunity); (7) Work Environment and Location (comfortable working conditions, location); (8) Work Itself (matching one’s interests, autonomy, independence, variety); and (9) Altruism/Contribution to Society (helping others, societal progress, meaningful nature of one’s work).

Some of these domains, such as Altruism/Contribution to Society, Work Itself and Organizational Culture and Management, were not incorporated in every scale, indicating that some scales might not be comprehensive enough. This tends to reflect the fact (as stated above) that different researchers have differing conceptions of work values and thus determine varying scale items. In general though, most researchers in Mainland China tend to conceptualize work values as criteria used for judging and selecting occupations, or what is important and desirable in vocational activities (cf., Cao 2003; Liu 2001; Luo and Luo 2006; Meng 2007; Ning 1996; Yang 2005; Yin, Dai, and Jin 2000; Yu and Huang 2000; Zhang 2005; Zhang 2007). Others, however, viewed work values as a personal belief system focusing on what is important for fulfilling personal needs (cf., Gu 2001; Tan and Yao 2005; Yu, Teng, Dai, and Hu 2004; Zhang 2007).

By comparison, researchers in the West embrace rather broader conceptions. For example, work values may be thought of as being “personal preferences for selected outcomes and rewards of working” (Rottinghaus and Zytowski 2006, p. 211), or “general attitudes regarding the meaning that an individual attaches to his [sic] work role” (Wollock *et al*. 1971, p. 331). Other interpretations include: “objectives (goals) that people seek to attain to satisfy their needs” (Super 1995, p. 54); “desirable end states realized through working” (Nord, Brief, Atieh, and Doherty 1990, p. 21); “generalized belief about the desirability of certain attributes of work” (Lyons, Duxbury, and Higgins 2006, p. 607); and “work aspects preferences” (Pryor 1981, p. 243).

## Work Values of University Students in Taiwan

Through the *Index to Taiwan Periodical Literature System* and by wider searching (e.g., Hung and Liu 2003), a few studies concerning the construction and utilization of localized work values scales among university students in Taiwan were identified. Again, quantitative and cross-sectional studies predominated, but inter-generational (Wang 1993) as well as in-depth qualitative studies on college students (Wang 1998; Yu 2007) and working adults (Chen and Liu 1995) were located. The key factors in the work values construct emerging from the identified reports are listed in Table 2.

**Table 2:** Factor-models derived from empirical studies of work values of university students in Taiwan

Huang, Wang, and Kuo (2006)	Wu, Lee, Liu, and O (2001)	Wang (1993)	Chen, Wang, Liu, O, and Lee (1987)
Economic Returns	Organizational Security & Economic Rewards	Extrinsic Rewards	Rewards
Self-Development	Self-Growth	Intrinsic Rewards	Self-Expression
Leisure & Welfare	Leisure, Health & Work Location	Extrinsic Rewards	Rewards
Future Outlook	Self-Growth	Intrinsic Rewards	Self-Expression
Work Atmosphere	Social Interaction	Security & Harmony	Work Environment
Security (work location & reputation of company)	Leisure, Health & Work Location	Security & Harmony	Work Environment
		Extrinsic Rewards	
Autonomy	Self-Esteem	Intrinsic Rewards	Self-Expression
Job Security	Stability & Anxiety Avoidance	Extrinsic Rewards	Rewards
	Organizational Security & Economic Rewards		
	Self-Esteem	Achievement	Achievement
	Self-Actualization (including Altruism)	Collective Interests & Benefits	Self-Expression Rewards
		***Instrumental values*** Stamina & Competency, Humility, Tolerance & Leniency, Contentment & Behaving Oneself, Justice & Self-Discipline, Pragmatism	

Common factors identified from the studies appear to be: (1) Economic Return/Extrinsic Return (pay, pension, leave, insurance); (2) Self-Growth/Self-Development/Self-Expression (suitably challenging work, higher-level of responsibility, training opportunity, creativity); (3) Leisure, Health, and Work Location (leisure activities, tours, personal health, convenient work location); (4) Future Outlook (work prospects, promotion); (5) Social Interaction/Work Environment (social relationship with co-workers and supervisors, work conditions); (6) Security and Harmony (job security, harmonious working relationships, inner peace); (7) Autonomy (autonomy, independence); (8) Collective Benefits/Altruism/Self-Actualization (national development, serving others, life goal, quality of life); and (9) Self-Esteem/Achievement (self-assurance, respect from others, sense of achievement). It can be seen that these nine domains encompass the six suggested by Leuty and Hansen (2011).

In passing, it should be noted that ‘Leisure’ seems to be a distinct factor identified in some Taiwanese studies (cf., Huang, Wang, and Kuo 2006; Wu, Lee, Liu, and O 2001). This factor never appears in Chinese Mainland research findings. Whether this implies that Taiwanese university students pay more attention to considering the importance of leisure (for maintaining a balanced lifestyle in relation to their future career choices) is unknown, but could be further investigated.

Wang (1993) developed a localized work values scale to measure Taiwanese university students’ ‘Terminal Values’ or ‘desirable end-states’ (e.g., Intrinsic Rewards, Extrinsic Rewards, Collective Benefits, Security and Harmony) and ‘Instrumental Values’ (e.g., Stamina and Competency, Tolerance and Leniency, Contentment and Behaving Oneself, Justice and Self-Discipline, and Pragmatism). The common factors listed above essentially belong to Terminal Values, while Instrumental Values fail to match with the key domains identified by Leuty and Hansen (2011) (see Table 2). This may be because these values are mainly based on Confucian orientation and were deliberately introduced by Wang (1993) as an attempt to follow the “Indigenization Movement” called for by the local researchers to better match the local culture and conditions. This indigenization of psychology in Taiwan started as early as 1976, with the aim of bringing to light both the universal and the unique psychological and behavioural characteristics of Taiwanese Chinese people (Yang 1997). The development of localized work values scales in Taiwan started from Chen, Wang, Liu, O and Lee’s (1987) study, based on translating and amending Donald Super’s *Work Values Inventory* (WVI) (Wu *et al*. 2001). The study by Hsia and Yau (1984) also focused on translation of the WVI into Chinese and its validation on local students. Such commendable efforts that seek to match research design and instruments to local conditions are certainly also necessary for studies involving other Chinese communities.

## Work Values of University Students in Hong Kong

Research has been carried out in Hong Kong on cultural values and work values using working adults; but there are very few studies of work values among university students. It also appears that there is still no localized instrument to measure work values in Hong Kong. The *PsycINFO database* yielded 40 results when searched in July 2011 using the keywords ‘work values’, ‘university students’ and ‘Hong Kong.’ Out of the 40 search results only 21 papers referred to scholarly journal articles. Closer examination found that among these articles only six were really concerned with Hong Kong, and none of these specifically studied work values of university students. Eventually, by searching more broadly, a few empirical studies were located (cf., Chow and Blumenfeld 1984; Cui *et al*. 2006; Fung 1979; So 1979).

The first published studies on work values of university students in Hong Kong started with the research projects of Fung (1979) and So (1979). The only study we could identify regarding the factor structure of work values of Hong Kong university students was published by Cui *et al*. (2006). Their study adopted a 32-item self-constructed work values scale, developed by the chief researcher (an academic in Mainland China). The study explored the structure of work values of graduating college students in Hong Kong and Mainland China through confirmatory factor analysis. It was found that the work values structure of Hong Kong students could be represented by three factors: (1) ‘Personal Values and Benefits’ (including items, such as ability utilization, relationship with co-workers, self-worth, mental health, good management, equitable opportunity, promotion, learning opportunity, working conditions, stability, personal development); (2) ‘Personal Development and Social Status’ (including items such as self-initiative, optimal level of stress, creativity, challenge, social status, and reputation); and (3) ‘Work Characteristics’ (including items such as annual leave, relaxing work, freedom, work location, flexibility, benefits, salary). The first two factors comprised both intrinsic motivational factors (e.g., realizing personal values in their jobs, promoting personal development) as well as extrinsic motivational factors (e.g., obtaining benefits, social status, and so on). In other words, Hong Kong students tended to consider both intrinsic and extrinsic factors simultaneously when contemplating job selection (Cui *et al*. 2006).

## Factor Structure of Work Values of Chinese University Students

Comparisons across the three locations (Mainland China, Taiwan and Hong Kong) reveal that the common factor structure of work values of Chinese university students tend to match the domains identified by Leuty and Hansen (2011) (see Table 3). However, two domains ― Family Maintenance (in Mainland China) and Wang’s (1993) Instrumental Values (in Taiwan) ― have no corresponding counterparts in the other regions. It should not be concluded from this that valuing Family Maintenance in relation to work values is a unique feature among university students in Mainland China. Quite clearly, strong family influences do exist in Taiwan as well, which is also a collectivist society under the influence of Confucianism. One possible explanation for this difference is that, despite attempts at indigenizing the construction of scales used in China and Taiwan, most are still based on Western theories of work values. For instance, the scale used by Wu *et al*. (2001) made reference to the models of Super, Elizur and Herzberg. Similarly, Chen *et al*. (1987) relied heavily on Super’s *Work Values Inventory*. These Western theories and instruments tend not to address issues of family support or family maintenance in relation to an individual’s work.

**Table 3:** Key domains of work values identified in the three locations compared to those identified by Leuty and Hansen (2011)

Leuty and Hausen (2011)	Chinese mainland	Taiwan	Hong Kong
Environment—physical condition of the workplace/quality of supervision/work-life balance/co-worker support	Interpersonal Relationship	Work Environment	Personal Values & Benefits
	Family	Security & Harmony	Work Characteristics
	Work Environment & Location		
Competence--- importance of challenging work/opportunity for competence/creativity/achievement/increased responsibilities/using one’s skills	Ability Utilization & Personal Development	Self-Development/Self-Growth/Self-Expression	Personal Values & Benefits
		Self-Esteem/Achievement	
Status--- status/prestige/high income/advancement opportunities	Welfare & Security for Material Rewards	Extrinsic Rewards	Personal Development & Social Status
	Status & Reputation	Future Outlook	Personal Values & Benefits
	Comfort Life		
Autonomy—the importance of having independence/responsibility over work tasks/variety	Work Itself	Autonomy	Work Characteristics
Organizational Culture--- moral/having fair company policies/support from management/proper training/clear procedures	Organizational Culture & Management	Work Environment	Personal Values & Benefits
	Work Environment & Location	Security & Harmony	
Relationships--- relationships with co-workers/helping others	Interpersonal Relationship	Security & Harmony	Personal Values & Benefits
	Organizational Culture & Management	Collective Interests & Benefits/Self-Actualization	
	Work Environment & Location		
	Altruism		
	***Family Maintenance***		
		***Instrumental values*** Stamina & Competency, Humility, Tolerance & Leniency, Contentment & Behaving Oneself, Justice & Self-Discipline, Pragmatism	

One of the fruitful outcomes from Taiwanese researchers’ indigenization efforts seems to be the emergence of Wang’s (1993) Instrumental Values. But again, this cannot be considered as a unique feature existing only among Taiwanese university students, and the validity of the Instrumental Values construct should be tested in other communities.

## Comparing East with West

Although we can say that the key domains of work values of university students are in general very similar between East and West, there seem to be subtle but important differences. For instance, ‘Relationship’ is a key value domain in both the West and East. In an individualistic society (typical of the West) good interpersonal relationships are important because they satisfy emotional needs and make people feel secure and contented, both at work and in life outside work. In a collectivistic society such as in Chinese contexts, ‘relationship’ (*“Guanxi”*) is more than just personal feelings and is related to membership of a social group, appropriate behaviours in a group (such as maintaining harmony among members) and even opportunity for getting access to resources. In the context of work, through having good relationships (*“Guanxi”*) with someone in power or in high status, an individual may gain significant benefits, including winning a job even though competitors may have higher ability or have performed better. That is the reason why ‘equitable opportunity’ and ‘fair competition’ become frequent items in the self-constructed work values scales in Mainland China. Equitable Opportunity is also one of the factors in the *Chinese Work Values Questionnaire* (CWVQ) (Zhang, Wang, Yang, and Teng 2007). In addition, ‘Relationship’ may include not only general interpersonal relationships, but also Family Maintenance.

Similarly, in both East and West, ‘Economic Returns’ (e.g., pay/salary) are prerequisite for a high standard of living and a comfortable life. For Chinese people, however, valuing economic returns does not merely reflect the tangible outcome of work but is closely related to the individual’s “*mianzi*” (self-respect, or ‘face’) in comparison with peers or other reference groups (Chu 2008). *Mianzi* is perceived as “an intangible form of social currency and personal status” and “is determined by social position and material wealth” (Luo 1997, p. 45). In Chinese collectivist societies an individual should have enough *mianzi* to present a favourable personal image to others of being competent in contributing to the social network (*guanxi*) (Humphreys 2007). Through *guanxi* one can often obtain favours or benefits that others who lack *guanxi* cannot. Not surprisingly, the importance of economic returns as a crucial job motivator is frequently reported by Chinese employees (Han and Kakabadse 2009).

It is because of such subtle cultural differences between West and East that researchers need to be sensitive to variations in the way that work is perceived and valued in different societies. In practical terms, instruments developed in the West may not contain specific items that help to identify unique culturally-influenced values in the East. The case for developing local scales remains strong.

## Conclusion

Career guidance and counselling services in universities in China, Taiwan and Hong Kong continue to expand in response to students’ needs and the realities of the job market. Well-focused research in domains such as work values has become a necessity, in order to provide information for personnel concerned with providing career services―in particular, data on students’ interests, motivations, attitude toward work, and factors that influence their career choices. For this reason, the construct of ‘work values’ should be explored further, using appropriate investigative tools in the Chinese context. This paper has reported some issues that have emerged already from studies in the East.

A search of the extant research literature has shown that the factor structure of work values of university students in Mainland China, Taiwan and Hong Kong essentially matches the key domains identified by Leuty and Hansen (2011). Only ‘Family Maintenance’ and Wang’s (1993) ‘Instrumental Values’ appear as unique to studies in the East. This tends to suggest a commonality of work values construct between the East and West; but at the same time, there are a few differences between cultures. It seems that instruments derived from Western models of work values may overlook some of these subtleties when applied in the East.

In spite of sharing the same cultural heritage, uniqueness in the three places can still be observed. Different foci or contents can be found in the work values scales under study, as shown in the examples of “Equitable Opportunity” and “Family Maintenance” (e.g., importance of residence permit) being particularly stressed in the People’s Republic of China, as well as Wang’s (1993) Confucian values and the importance university students attached to leisure in Taiwan. These stand-out values may reflect specific contextual and social phenomena of these places, and thus disprove the assumption that the work values construct is the same for all Chinese societies. For this reason, we argue the need for localized scales and contextualized measures that can better capture the relevant components of work values for a particular society. This seems to be particularly urgent for Hong Kong, where there are currently no indigenous scales for assessing work values.
